# Use of Three-Dimensional Computed Tomography to Classify Filling of Alveolar Bone Grafting

**DOI:** 10.1155/2012/259419

**Published:** 2012-11-06

**Authors:** Antonio Jorge V. Forte, Renato da Silva Freitas, Nivaldo Alonso

**Affiliations:** ^1^Section of Plastic and Reconstructive Surgery, Yale University School of Medicine, New Haven, CT, USA; ^2^Division of Plastic and Reconstructive Surgery, Federal University of Paraná School of Medicine, 81.050-000 Curitiba, PR, Brazil; ^3^Division of Plastic and Reconstructive Surgery, University of São Paulo School of Medicine, São Paulo, SP, Brazil

## Abstract

Several authors have proposed classifications to analyze the quality over time of secondary alveolar bone grafting. However, little discussion has been held to quantitatively measure the secondary bone grafting for correction of nasal deformity associated to cleft palate and lip. Twenty patients with unilateral alveolar cleft, who underwent secondary alveolar bone grafting, were studied with 3D computer tomography. The height between the inferior portion of the pyriform aperture and the incisal border of the unaffected side (height A) and the affected side (height B) was measured using a software Mirror. A percentage was then obtained dividing the height B by the height A and classified into grades I, II, and III if the value was greater than 67%, between 34% and 66%, or less than 33%. Age, time of followup, initial operation, and age of canine eruption were also recorded. All patients presented appropriate occlusion and function. Mean time of followup was 7 years, and mean initial age for operation was 10 years old. 16 patients were rated as grade I and 4 patients as grade II. No cases had grade III. We present a new grading system that can be used to assess the success of secondary bone grafting in patients who underwent alveolar cleft repair.

## 1. Introduction

After initial cleft lip and palate repair, the residual bony defect is addressed with secondary bone grafting. This approach carries the following advantages: (1) maxillary stabilization; (2) effective closure of oronasal fistulae; (3) better support for the defective alar base, reducing nasal asymmetry and impairment of facial contour; (4) faster malocclusion correction with orthodontic treatment [1, 2]. Studies showed that the gap in the dental arch was closed orthodontically in 90%, and it was proposed that the ideal time for secondary bone graft is between 9 and 11 years of age [1, 2]. Abyholm et al. were the first to suggest the use of radiographic measure of interalveolar septum height as a grading system, which consisted of type I (height approximately normal), type II (at least 3/4 of normal height), type III (less than 3/4 of normal height), and type IV (failure), which was popularized by Bergland et al. in 1986, also known as the Oslo grading system [1]. Since then, multiple scales to assess the success of secondary alveolar bone graft have been proposed [1–4]. Kindelan et al. proposed the use of a 4-point scale that measured the degree of bony filling in the cleft area when compared to its initial bone graft site, which was radiographed after orthodontic treatment and prior to surgery. The scale ranged among grade I (more than 75% bony filling), grade II (50 to 75% bony filling), grade III (less than 50% bony filling), and grade IV (no complete bony bridge). Their scale seemed to be reliable and showed moderate to substantial intraobserver agreement and fair-to-moderate interobserver agreement [4]. Hynes and Earley proposed, in 2003, a modification for the Oslo grading system. Their mean followup between grafting and radiographic assessment was 4.5 years. They performed a 3 × 4 cm periapical dental radiograph. The occlusal level, the basal level, and the total height of the newly acquired bone in the alveolar cleft were graded using the Oslo system, and the bone graft height was compared with the expected height of normal interdental alveolar bone in corresponding films. Long Jr et al. studied contours of the grafted bone of 46 cleft sites, with a mean follow-up time of 3.1 years. They established a series of ratios of measurements obtained directly from the radiograph and were able to detect failure of the formation of a bony bridge. The measurements included the amount of notching of the bone graft, the length of the proximal and distal segment anatomic root, the location of the alveolar crest, and the size of the most coronal attachment of the bone bilaterally [5]. Witherow et al. analyzed radiographs of 87 cleft sites using an 8-point scale to describe position of bone graft after secondary alveolar grafting in relation to the cleft roots, and their scale can also be used in mixed dentition, as long as the roots can be divided into four, and the radiograph is directed through the cleft line. In addition, depending on the positions of the bony bridge across the cleft, the X-rays were classified into one of six groups (A to F) [6]. Nightingale et al. compared three methods for radiographic analysis proposed by Bergland et al, Kindelan et al., and Witherow et al. [2, 4, 6]. They found that none of the three radiographic scales showed superior reproducibility over the other two, and that each scale seemed to be more reproducible in the mixed dentition, that neither occlusal nor periapical X-rays were found to be more useful in assessing alveolar bone grafting success [7]. 

 At this time, the traditional scales lack valuable information of how well the graft takes in the area between the incisal border and the inferior border of the pyriform aperture, an important region that constitutes the bony base for the nasal alae (Table 1). For this reason, our goal is to propose a new grading system that can be used to assess the success of secondary bone grafting, at the level of the pyriform aperture, in patients who underwent alveolar cleft repair.

## 2. Method

**Figure 1:**
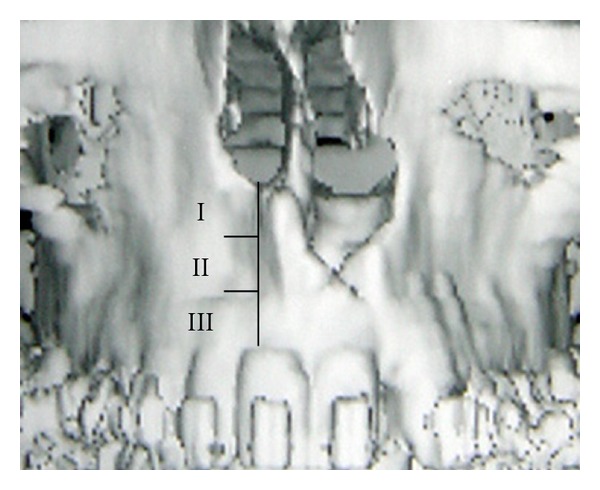
Classification based on pyriform aperture. Grade I: 67–100%; grade II: 34–66%; grade III: 0–33%.

Twenty patients with unilateral alveolar clef underwent secondary alveolar bone grafting by a single experienced craniofacial surgeon using the same technique. They were studied with three-dimensional computed tomography. The height between the inferior portion of the pyriform aperture and the incisal border of the good side (height A) and the affected side (height B) was measured using a software Mirror (Figure 1). A percentage was then obtained dividing the height B by the height A and classified in grades I, II, and III if the value was greater than 67%, between 34 and 66%, or less than 33% (Table 2). Age, time of followup, initial operation, and age of canine eruption were also recorded.

## 3. Results

**Figure 2:**
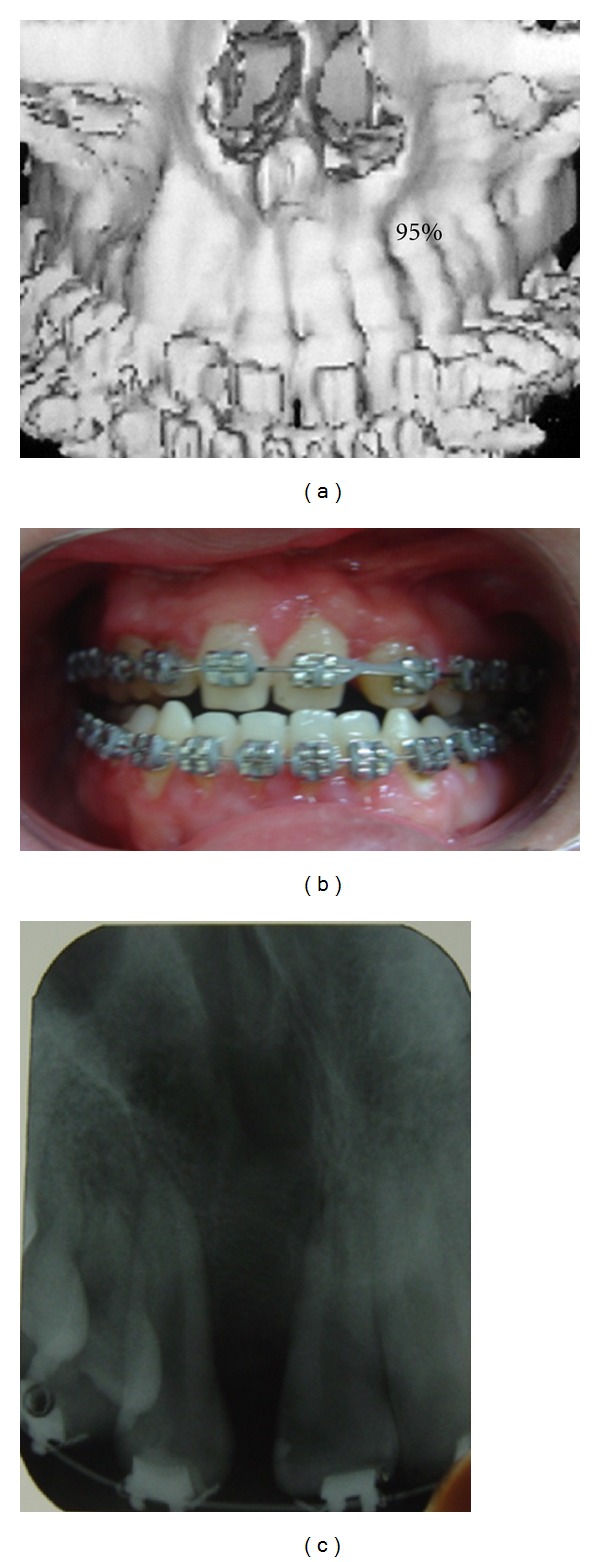
Patient with grade I. (a) CT scan view; (b) occlusal view; (c) dental X-ray.

**Figure 3:**
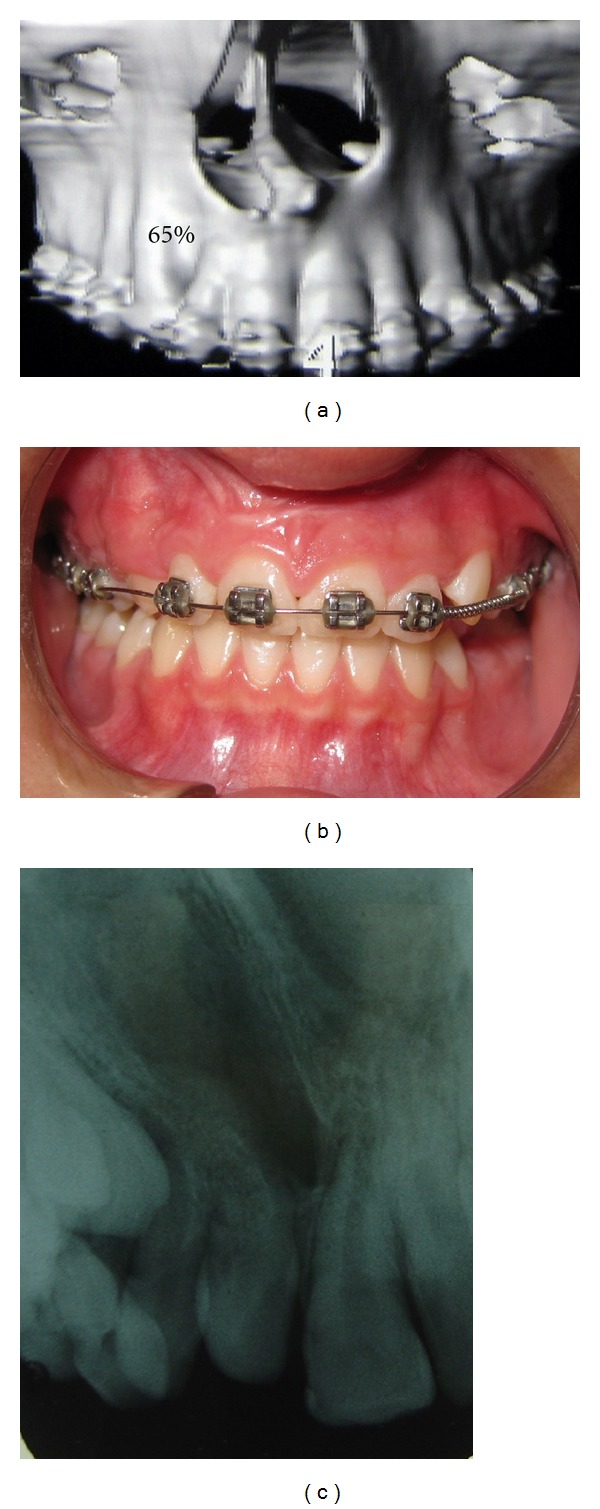
Patient with grade II. (a) CT scan view; (b) occlusal view; (c) dental X-ray.

All patients presented appropriate occlusion and function. Mean time of followup was 7 years, and mean initial age for operation was 10 years old. Sixteen patients were rated as grade I (Figure 2), and 4 patients as grade II (Figure 3). No cases had grade III.

**Figure 4:**
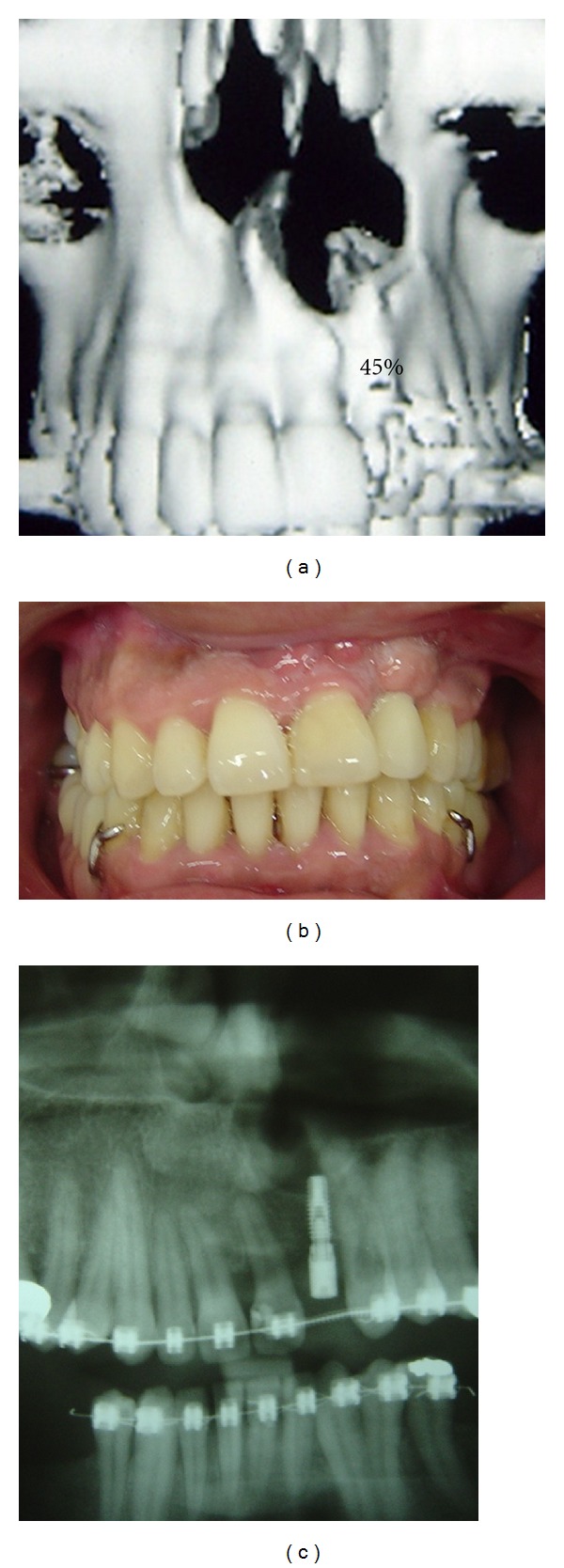
Patient with grade II. (a) CT scan view; (b) occlusal view; (c) panorex demonstrating dental implant.

Interestingly, for the patient with grade II and partial result on the pyriform aperture, as demonstrated in Figure 4, it was still possible to perform dental implant in the area. In the other classifications, this case would be considered successful.

## 4. Discussion

The development of three-dimensional computed tomography enabled a better appreciation of volume that conventional two-dimensional plain radiographies are unable to provide. Feichtinger et al. prospectively studied twenty-four patients with complete unilateral cleft of lip and palate, measuring the cleft defect and bone bridges with three-dimensional computed tomography three years after the secondary alveolar bone graft with iliac crest. They concluded that conventional two-dimensional radiograph underestimates the amount of bone resorption in transversal dimension when compared to three-dimensional computer tomography [8]. CT offers better image quality and accuracy without anatomic superimposition when compared with traditional X-rays [9]. 

Arctander et al. suggest that one should graft as much bone as possible to obtain adequate facial appearance. Their study examined 18 patients with complete unilateral cleft lip and palate using computed tomography 20 years after secondary cancellous bone graft from the iliac crest. They concluded that, even though all dental gaps were closed and patients were functionally intact, the amount of alveolar bone in the cleft side was less than that of the noncleft side [10]. Feichtinger et al. also showed that absence of adjacent teeth to the cleft site leads to mean bone volume loss of 95% [11].

Bergland et al. believe that nasal asymmetry, which is partially caused by skeletal malformation, can be to some degree corrected with filling in the alar base with cancellous chips [2]. The use of the current scales to measure bone graft size lacks valuable information regarding the portion of the graft that aims at correction of the pyriform aperture deformity and subsequent increase in nasal projection. Therefore, appropriate assessment of the area between the incisal border and the inferior border of the pyriform aperture is needed. Our study shows that the traditional grading systems classify as complete success bone grafts regardless of how properly they correct the nasal bone structure misshapenness. Hence, we would like to suggest the association of the traditional classifications and this new one in order to better evaluate the correction of facial bone structures affected by the cleft abnormality. Therefore, grade I would be considered success, grade II would be considered partial success, and grade III would require repeat procedure in future. In our experience, grafting success is best measured once maxillofacial growth is completed. It is also important to notice that this method is subject to limitations, such as asymmetrical pyriform aperture, dental crowding, and bilateral clefts. Also, this classification can be used even in cases of malocclusion, as long as the malocclusion is not caused by gross deformity of the dental arch around the canines. 

In conclusion, we would like to propose a new grading system that can be used to assess the success of secondary bone grafting in patients who underwent alveolar cleft repair, using three-dimensional computed tomography and the inferior portion of the pyriform aperture as a bony landmark.

## Figures and Tables

**Table 1:** Literature review of major publications involving description of grading system to assess secondary bone grafting.

First author	Journal	Grading system
Abyholm 1981 [1]	SJPRS	Radiographical measurement of interalveolar septum height as a grading system: type I (height approximately normal), type II (at least 3/4 of normal height), type III (less than 3/4 of normal height), and type IV (failure)
Bergland 1986 [2]	CPJ	Popularized the Oslo grading system, which is described above
Long Jr 1995 [5]	CPCJ	Studied contours of the grafted bone, using ratios. The measurements included the amount of notching of the bone graft, the length of the proximal and distal segment anatomic root, the location of the alveolar crest, and the size of the most coronal attachment of the bone bilaterally
Kindelan 1997 [4]	CPCJ	4-point scale that measured the degree of bony filling in the cleft area when compared to its initial bone graft site. Grade I (more than 75% bony filling), grade II (50 to 75% bony filling), grade III (less than 50% bony filling), and grade IV (no complete bony bridge)
Witherow 2002 [6]	CPCJ	8-point scale to describe position of bone graft after secondary alveolar grafting in relation to the cleft roots. Depending on the positions of the bony bridge across the cleft, the X-rays were classified into one of six groups (A to F). May be used with mixed dentition.
Hynes 2003 [3]	BJPS	Modification for the Oslo grading system using periapical dental X-ray. The occlusal level, the basal level, and the total height of the newly acquired bone in the alveolar cleft were graded using the Oslo system, and the bone graft height was compared with the expected height of normal interdental alveolar bone in corresponding films

Journals: BJPS: British Journal of Plastic Surgery; CPCJ: Cleft and Palate Craniofacial Journal; CPJ: Cleft Palate Journal; SJPRS: Scandinavian Journal of Plastic and Reconstructive Surgery.

**Table 2:** Description of new grading system to assess secondary bone grafting.

Grade	Percentage affected/unaffected side
I	Above 67%
II	34–66%
III	33% or less
